# A culturally adapted brief intervention for post-traumatic stress disorder in people with severe mental illness in Botswana: protocol for a randomised feasibility trial

**DOI:** 10.1186/s40814-021-00904-1

**Published:** 2021-09-03

**Authors:** Keneilwe Molebatsi, Lauren C. Ng, Bonginkosi Chiliza

**Affiliations:** 1 Department of Psychiatry, Nelson R. Mandela School of Clinical Medicine, University of KwaZulu-Natal, Durban, South Africa; 2 Department of Psychiatry, Faculty of Medicine, University of Botswana, Private Bag, 00713 Gaborone, Botswana; 3 Department of Psychology, University of California Los Angeles, Los Angeles, CA USA

**Keywords:** Trauma, Psychological intervention, Cultural adaptation, Psychoeducation, Feasibility, Acceptability

## Abstract

**Background:**

Research consistently reports elevated rates of exposure to traumatic events and post-traumatic stress disorder (PTSD) in people with severe mental illness (SMI). PTSD may be adequately managed with psychotherapy; however, there is a gap when it comes to management in culturally diverse settings like Botswana. This paper describes a study protocol whose aim is to culturally adapt the BREATHE intervention, a brief psychological intervention for people living with comorbid PTSD and SMI that was developed and tested in the USA; assess the feasibility and acceptability of the adapted BREATHE intervention and explore its efficacy.

**Methods:**

The study will be conducted in three phases using a mixed methods approach. The first phase will identify and describe the most common traumatic experiences and responses to traumatic experiences, amongst patients with SMI, and patients’ and mental health care providers’ perceptions about suitable PTSD interventions for Botswana. The second phase will entail cultural adaption of the intervention using findings from phase 1, and the third phase will be a pilot trial to assess the feasibility and acceptability of the culturally adapted intervention and explore its efficacy. Quantitative and qualitative data will be analysed using basic descriptive statistics and thematic analysis, respectively.

**Discussion:**

Literature highlights cultural variations in the expression and management of mental illness suggesting the need for culturally adapted interventions. The findings of this feasibility study will be used to inform the design of a larger trial to assess the efficacy of an adapted brief intervention for PTSD in patients with SMI in Botswana.

**Trial registration:**

Clinicaltrials.gov registration: NCT04426448. Date of registration: June 7, 2020.

## Background

Patients with severe mental illness (SMI) are at an increased risk of exposure to trauma compared to the general population [1, 2]. Surveys of the general population have reported that 70.4% of the participants had been exposed to traumatic events [3] compared to the almost universal (98%) trauma exposure in patients with SMI [2]. The elevated rates of trauma exposure result in a high prevalence of PTSD in this population, for example, Mueser et al. reported prevalence rates of ranging between 29 and 43% which is significantly higher than the estimated rates of 7 to 12% in the general population [4].

Psychiatric hospitalisation and childhood trauma are significant contributors to the high rates of PTSD in this population. Aspects of psychiatric hospitalisation such as physical restraint, use of seclusion, arraignment by police, involuntary admissions and being around violent patients have been described by patients as traumatic [2, 5]. In addition, childhood trauma has been reported to be highly prevalent amongst patients with SMI; the neurodevelopmental effects of childhood trauma increase vulnerability to negative outcomes of exposure to trauma such as development of PTSD [6].

The most used trauma focused psychological interventions are cognitive behavioural therapy, prolonged exposure and eye movement desensitisation and reprocessing therapy [7, 8]. These interventions may not be feasible in Botswana because they require specialised mental health services which are a limited resource in the country. Currently, there are 11 psychiatrists in the country, of which five are academics, three are in private practice and the rest are in public service. There is one referral hospital in the country which has two qualified clinical psychologists.

The Brief Relaxation, Education And Trauma HEaling (BREATHE) is a three session evidence-based intervention for PTSD in patients with SMI. It can be administered by trained lay personnel. BREATHE is an appropriate intervention to be used in Botswana because suitable interventions in resource-limited settings need to have demonstrated effectiveness in alleviating symptoms, should not require specialised mental health training and should have few sessions to reduce attrition rates [9]. Evidence from previous research has demonstrated cultural variation in the experience and explanation of mental health symptoms; we therefore hypothesise that the BREATHE intervention will require cultural adaptation to localise the interpretation and understanding of trauma. Adaptation addresses the mismatch between the population, setting and culture in which the evidence-based intervention was validated and the target population, setting and culture in which it will be used. Adapted interventions are likely to be more acceptable, effective and feasible [10, 11]. Cultural adaptation of evidence based intervention may be achieved by translation to local languages and incorporation of culture specific norms and beliefs [12].

This protocol describes a study whose aim is to culturally adapt the Brief Relaxation, Education and Trauma Healing (BREATHE) intervention, explore its efficacy and examine the feasibility and acceptability of the culturally adapted intervention in the treatment of post-traumatic stress disorder amongst patients with severe mental illness in Botswana.

## The intervention

The BREATHE intervention is an evidence-based, digital video-based intervention for PTSD in patients with SMI which was developed in the USA [13]. The programme comprises education about trauma and PTSD, symptoms of PTSD, and educates patients on self-management of anxiety by breathing retraining. BREATHE has been shown to significantly reduce PTSD and depression symptoms with the effect maintained up to 3 months post treatment [13]. The components are done in three sessions thereby reducing the likelihood of dropouts encountered in other psychological PTSD interventions which require more sessions.

BREATHE therapy discussions are guided by a pre-recorded digital video (DVD). The videos discuss the following: (i) potential traumatic events that are commonly experienced in the US population; (ii) symptoms that individuals may experience after being exposed to potential traumatic events and (iii) information about PTSD. Patients are given handouts and worksheets to complete at home. For example, at the end of the first session in which the clients are taught breathing retraining, the client is given a handout to document breathing retraining practice at home.

## Methods

The study will be conducted in three phases. The first phase will collect formative data to inform the adaptation of BREATHE, including the perceptions of mental health clinicians (doctors, nurses, clinical psychologists and social workers) about trauma experiences amongst their clients (patients with SMI) and about the BREATHE intervention. The second phase will utilise the findings from phase 1 to culturally adapt the BREATHE intervention to the local context. In the third phase, a randomised pilot trial will be conducted to examine the feasibility and acceptability of the culturally adapted BREATHE intervention amongst patients with SMI in Botswana, and explore its efficacy.

### Study setting

Data will be collected at Sbrana Psychiatric Hospital (SPH) which is situated in Lobatse, in the South Eastern part of Botswana. SPH is the only referral psychiatric hospital in Botswana. Services offered at SPH include outpatient and inpatient psychiatric treatment and care, forensic assessment, occupational therapy and rehabilitation, psychotherapy, substance use disorders management and nutrition and dietetics services.

Patients referred to SPH are those with SMI and are largely representative of the culturally diverse Botswana population, thus making it the best setting to conduct this study.

### Phase 1. Understanding trauma in context

#### Study design and participants

An exploratory mixed methods study design [14] will be used in this phase. Individual in-depth interviews and quantitative surveys will be conducted with patients with SMI to determine the most common types of traumatic events and post-traumatic symptoms, as well as perceptions of the proposed intervention. The World Health Organisation (WHO) classifies schizophrenia and related conditions, bipolar disorder and moderate to severe depression as severe mental disorders [15]. Patients with these diagnoses will be classified as ‘patients with SMI’ in this study. Focus groups will be conducted with mental health clinicians based at SPH to explore perceptions towards traumatic experiences amongst patients they care for, interventions they use or know about for the management of PTSD and the proposed BREATHE intervention.

#### Sample size and sampling

Creswell (2007) recommends a minimum of five participants and a maximum of 25 participants for a phenomenological study [16]. Using these guidelines, a sample size of 15 patients with SMI has been set; however, the final sample size will be dependent on data saturation; whereby researchers note that there is no new information or themes being gained from more interviews [17]. A minimum of four focus groups of eight mental health clinicians in each group will be conducted. Six to eight participants have been recommended as an optimum number in focus group discussions because there is less risk for limited discussions seen with low numbers, or a chaotic unmanageable group seen with high numbers of participants.

Purposeful sampling will be used to select mental health clinicians and patients with SMI. The sampling method involves identification and selection of participants knowledgeable about or those who experienced a phenomenon of interest [18]. For patients to be enrolled in the first phase of the study they should be at least 18 years old, able to understand Setswana or English and must meet the DSM-5 criteria for any SMI as categorised for this study. Both inpatients and outpatients will be recruited into the study. For patients whose SMI diagnosis was made by a medical officer, they will be screened by a qualified psychiatrist to establish the diagnosis using DSM-5 diagnostic criteria [19]. Focus groups will be characterised by heterogeneity: mental health clinicians with variation in age, gender, qualification and profession amongst participants to allow for contrasting opinions. Mental health clinicians who will be invited to participate in the focus groups should have worked in Sbrana Psychiatric Hospital for a minimum of 6 months and must be involved in the direct care of patients with SMI.

### Measures

#### University of California, San Diego Brief Assessment of Capacity to Consent (UBACC) [20]

The 10-item instrument offers a comprehensive evaluation of understanding and appreciation of the study components. It therefore allows researchers to note that enrolled participants had a basic understanding of the study elements deemed essential to giving an informed consent. The UBACC can be administered by trained research staff with bachelor’s degree–level qualifications. Items are scored on a scale of 0 to 2 points, with 0 reflecting a clearly incapable response and 2 indicating a clearly capable response. Principal investigators prepare answers that receive scores of 0, 1 or 2 before commencing recruitment, and research assistants are allowed to explain or paraphrase the UBACC questions if a potential participant seems not to understand. A cut-off score of 14.5 out of a maximum total of 20 has been selected based on the receiver operating characteristic curve analysis of the UBACC [20]. The UBACC has been used previously in an African population with SMI where it proved to be an effective tool to improve understanding of research study elements during consent, and the authors concluded that the UBACC may be particularly important in groups with SMI [21, 22].

#### The Childhood Trauma Questionnaire-Short form (CTQ-SF) [23]

The CTQ-SF is a 28-item self-report measure of abuse and neglect in childhood. Respondents endorse childhood experiences on a 5-point Likert scale ranging from 1 (never true) to 5 (very often true). The CTQ consists of five subcategories, three assess abuse (physical, sexual and emotional) and two assess neglect (emotional and physical). Each subscale score ranges from 5 (no history of abuse or neglect) to 25 (very extreme history of abuse and neglect). The CTQ-SF has demonstrated good internal consistencies for the scale and subscales (*α* = 0.7-0.9); the 4-week test–retest reliability of the CTQ was also good at Spearman’s *ρ* = 0.75 when used in a SMI population suggesting that the CTQ is not likely to be influenced by reporting biases associated with shifts in mood or level of psychological distress [24]. The CTQ-SF has been used amongst populations with SMI in South Africa [25, 26].

#### The Life Events Checklist for DSM-5 (LEC-5) [27]

The most widely used self-report instrument which screens for exposure to traumatic experiences [28]. The LEC-5 comprises 16 events which have been found to result in distress and increase risk of developing PTSD. The last question (number 17) enquires about any other very stressful event or experience not included in the first 16 items. Respondents indicate varying forms of exposure (happened to me; witnessed it; learned about it; part of my job; not sure; doesn’t apply) to each type of potentially traumatic event included on a 6-point nominal scale, and respondents may endorse multiple levels of exposure to the same trauma type. The instrument has been used in studies conducted in Southern Africa including South Africa [29, 30] and Zimbabwe [31]. Additional items developed from findings of a survey amongst an Ethiopian population with SMI [32] will be added to the LEC and edited as new items emerge during the interviews. Examples of the additional items include the following: someone restrained, chained or forced to be alone or isolated; exploitation, such as property being stolen, or someone intentionally not being paid for work.

#### Zulu Culture Specific Trauma Experience Questionnaire (ZTEQ)

Is a culture-specific tool that was developed to enhance the assessment of trauma and increase the rate of eliciting a history of traumatic events amongst a Zulu population*.* The instrument was found to increase the probability of eliciting traumatic events by 19.4% [33]*.* The Z-CTEQ increases the probability of eliciting traumatic events in an African population because it enquires about the role of witchcraft and ancestral displeasure in the causation of potentially traumatic events; these are not included in other instruments developed in Western countries*.* Botswana have the same cultural beliefs; therefore, the instrument is suitable for use in Botswana.

The instruments will be translated from English to Setswana using the WHO process of translation and adaptation of instruments [34]. The process includes the following steps: (i) forward translation by a bilingual translator; (ii) back translation by another independent bilingual translator; (iii) solving discrepancies between the versions by consensus between the translators and an expert panel of three mental health clinicians (psychiatrist, social worker and clinical psychologist); (iii) pre-testing and cognitive interviews amongst a population similar to end users of the instruments (patients with SMI).

### Data collection procedures

#### Patient interviews

Potential participants will first be guided through the UBACC and those who score 14.5 points or more on the UBACC will be invited to participate in the study. Only those who give consent will be included in the study. Participants who cannot write will be invited to ‘sign’ the consent forms with an X. Those who decline to sign the consent form will be excluded from the study. They will be led through a researcher-designed demographic questionnaire, CTQ-SF and the LEC-5, by the first author. The measures will be used to give us an understanding of some of the traumas that the participants have experienced through their lifetime. They will also be useful as we will be able to refer to these traumas during the interviews as we seek more detail and clarity on their experiences.

After administration of the measures described above, the first author will conduct in-depth semi-structured interviews with each participant. Demographic and clinical information such as gender, age and diagnosis will be obtained from the patient and corroborated with patients’ hospital records.

After general questions to corroborate data from the hospital records, patients will be asked to define trauma in their own words (for example: *Can you tell me what the word ‘trauma’ means to you?*). After the description, patients will then be asked if they have ever experienced anything, they considered traumatic (*Have you ever considered an event or experience that you or another person has experienced to be traumatic*? *The event could have happened to you or a close family member or friend, or you could have witnessed it happening to someone else*). The LEC-5 [27] with additional items [32] and the CTQ-SF [23] will be used to guide the interviews on potentially traumatic experiences that participants may have been exposed to. We will utilise some of the screening questions from the Zulu Culture Specific Trauma Experience Questionnaire (ZTEQ) to enhance understanding of culturally specific trauma experiences such as having a frightening experience which the participant believes was a result of failure to fulfil traditional rituals, displeasure by ancestors or witchcraft [33]. The participant’s description of trauma will be used in the analysis of the data to develop an understanding of how Botswana patients with SMI describe trauma.

To further understand trauma and its consequences amongst Botswana patients, the interview will enquire about reactions and symptoms experienced after the trauma exposure. Participants will be asked how the post-traumatic reactions affected their lives (for example: *In what ways, if at all, do these reactions interfere with your ability to work, interact with others or engage in your day-to-day activities?*) and how they have been coping after being exposed to the traumatic event (*What do you do to help yourself when you are experiencing these reactions? Where do you go for help when these things happen?*). The Post Traumatic Checklist-5 [35] will be used to probe for specific symptoms of PTSD.

The researcher will also find out if participants who have been exposed to traumatic events are interested in treatment and what preferred treatment would look like. A brief introduction about BREATHE will be done. The researcher will briefly describe to individual patients the basics of the intervention and share a clip of a video from the original intervention which will be translated to Setswana for those who do not understand English. Participants will then be asked to say their opinions about the intervention such as ‘What do you think about the intervention I just described?’ Probes will include: ‘What do you like about it?’, ‘What do you not like about it?’, ‘What would make it better?’.

#### Clinician focus groups

Mental health clinicians will be recruited conveniently from Sbrana Hospital outpatients and inpatients hospital facilities. Recruitment for the focus groups will be done over a period of 1 month so that nurses on night shifts and leave may be approached when present during day shifts. Only those who give written consent will be included in the focus groups.

The focus groups will be held at a time and location convenient to the mental health clinicians. We aim for each group to have discussions over a duration of 90-120 min. Focus groups will aim to find out whether mental health clinicians consider trauma to be an important consideration in the management of patients with SMI and whether they perceive BREATHE to be feasible after being shown a clip of the original video. Questions about BREATHE will be asked after a short information session about the intervention. Interview guides will include questions such as the following: ‘is trauma an important issue in your setting/amongst patients with SMI that you care for?’, Do you think this kind of intervention could work in Botswana?’, ‘What would make it easier?’, ‘Would such an intervention be acceptable to your patients?’, ‘What would make it more acceptable?’.

### Data analysis

Quantitative data will be analysed using basic descriptive statistics to describe the types of trauma experienced by the participants. Audio-recorded interviews will be transcribed verbatim and then thematic analysis [36] will be conducted. Data will be coded and reduced to themes to understand a description of traumatic experiences, conceptualise PTSD symptoms amongst patients with SMI; understand perceptions of mental health clinicians towards traumatic experiences and PTSD symptoms amongst patients with SMI in Botswana and perceptions towards the BREATHE intervention.

### Phase 2. Cultural adaptation of the BREATHE intervention

#### Study design

**Fig. 1:**
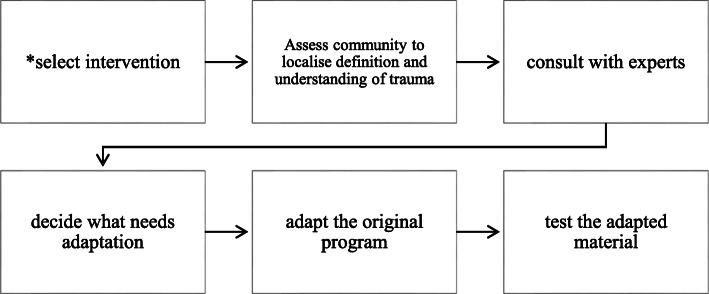
Key adaptation steps (adapted from [34]). Key: Asterisk indicates completed step

Steps outlined by Escoffery and colleagues [37] will be used to culturally adapt the BREATHE intervention. Figure 1 illustrates steps that will be followed.

#### Adaptation procedure

A community working group will be formed to give contextual, cultural and clinical insight in the process of adaptation. The group will comprise conveniently selected experts in trauma management such as clinical psychologists and psychiatric social workers involved in assessment, diagnosis or treatment of patients who present with mental health concerns. The researchers aim to include a different sample of mental health clinicians from the ones interviewed in phase 1. The purpose of the community working group will be to decide which components of the intervention require adaptation and how, based on the themes that were identified from the qualitative data collected in phase 1.

The community working group will work collaboratively with the researchers to adapt the BREATHE treatment manual, materials and handouts to the target population and setting, using results from the qualitative data. For example, one of the anticipated adaptations is the need to recreate the BREATHE psychoeducation video vignettes using culturally appropriate scenarios, characters, local idioms and descriptions obtained from analysis of phase 1 data. The community working group and the researchers will work together to formulate scripts that will be used by actors to film simulated culturally adapted patient-therapist scenes that provide the same psychoeducation content and sequence as the original BREATHE video vignettes [13]. Before filming, the actors will present the scripts to patients for feedback and the researchers and the community working group will review the script to ascertain fidelity to the original intervention video.

To complete adaptation of the intervention, The standard “forward-backward” translation procedure recommended by WHO will be used [34] to translate the BREATHE manual, work sheets, patient handouts and outcome measures to Setswana. The process includes forward translation, expert panel back-translation and pre-testing or cognitive testing.

### Phase 3. Pilot trial of the culturally adapted BREATHE intervention

#### Study design

A mixed methods approach [38] will be utilised for this phase. Quantitative research methods will be used to (i) explore the efficacy of the BREATHE intervention for the management of patients with comorbid PTSD and SMI, (ii) to assess the impact that BREATHE will have on participants’ knowledge of PTSD and (iii) to determine the effect of BREATHE on trauma, depressive and anxiety symptoms. In-depth individual interviews with participants will be used to explore patients’ experiences and perceptions regarding the intervention.

We will also conduct a physiological assessment of arousal associated with trauma symptoms using an eSense EDA skin conductance device. Individuals with PTSD have been shown to have greater skin conductance compared to those without PTSD [39].

#### Participants

Before patients who have consented to participate in the study sign an informed consent form, the UBACC which would have been adapted for the pilot trial will be administered. Only those who score 14.5 out of the maximum score of 20 will be allowed to participate. For patients to be eligible to participate in the pilot trial, they should be at least 18 years old, able to understand Setswana or English, must meet the DSM-5 criteria for any SMI as categorised for this study and meet the criteria for post-traumatic stress disorder as assessed with the Post Traumatic Checklist-5 (PCL-5), and the MINI PLUS PTSD module. Patients on psychotropic medication should have been on a maintenance dose for a minimum of 1 month and should not be undergoing trauma focused psychotherapy. Participants should be able and willing to come to the hospital for the period of data collection. Participants found to have an acute positive screen on the Ask Suicide-Screening Questions (ASQ) [40] will be excluded from the study as that requires urgent intervention.

#### Sampling and sample size

A pilot study is used to examine the effects of an intervention on clinical outcomes, get an understanding of the process of the intervention, feasibility and possible facility barriers [41, 42]. The current flat rule of the thumb approves 12 participants per group during a pilot study as an acceptable sample size [43]. Van Belle [44] has also proposed the same recommendation. Participants for the pilot trial will be purposively selected; recruiting only patients who have been diagnosed with SMI and PTSD. The total sample size for the pilot trial will be 40 to accommodate dropouts. There will be a group that will be randomised to receive the intervention and a group randomised to the control condition who will receive treatment as usual which is described under the study procedure. Each group will have 20 participants.

#### Data collection procedures

Before intervention delivery, participants who meet the inclusion criteria will be randomly placed into two groups using a web-based randomisation system on https://www.randomizer.org/. Outcome assessors will be blinded to the allocation. KM will assign participants based on the outcomes of the web-based randomisation system.

##### Intervention group

The adapted BREATHE intervention manual will be utilised in the delivery of the three sessions of the intervention [45].

Session 1 begins with an introduction of the intervention to the participant which will be followed by helping the patient/participant identify their most traumatic experience. With the aid of the culturally adapted digital video (DVD), discussions about experiencing trauma in the context of the index traumatic event/s and consequences follow. Lastly, breathing training will be taught by demonstration by the clinician and practice by the client. The client will be given a handout to document breathing retraining practice at home. The client is encouraged to practice daily until the next session, and in situations where they feel calm and safe in order to develop the skill.

Session 2 begins with setting an agenda for the session, this will help the client to know what to expect during the session. A review of the previous session and breathing retraining homework then ensues. Additional training is done as indicated. Psychoeducation about the development of PTSD and PTSD symptoms with reference to patient’s symptoms is then provided. The discussion is guided by the use of a video. The session ends with homework for the client to continue daily breathing retraining exercises and completion of worksheets related to information about PTSD.

Session 3 starts with a review of homework and agenda setting. The client is then educated on problems associated with PTSD such as substance use disorders, depression and interpersonal relationship difficulties. The discussion will focus on problems experienced by the client. Before the end of the session, the programme is wrapped up, reviewed and terminated. The client is offered continued support should they need it.

##### Treatment as usual

Standard usual care for patients with comorbid PTSD and SMI in Botswana concentrates on managing the SMI. Patients with SMI are rarely screened for symptoms of PTSD and as a result never receive any intervention for PTSD. If patients who have been enrolled for the trial are started on medication or psychotherapy, the treatment will be noted to adjust for confounding.

##### Dropouts

At recruitment, patients and caregivers’ contact details will be collected. An alternative date of appointment will be set with patients who do not show up or those who report that they will not make it for psychotherapy on the set date. Caregivers will be contacted if patients miss two appointments. Patients who miss three consecutive appointments will be recorded as treatment dropouts. The research team will follow up dropouts to collect final assessment data at a place convenient and agreeable to the participants.

#### Quantitative assessments of clinical symptoms, and functioning

Due to the small sample size and limited financial and time resources, validation of the instruments will not be done. The researcher will conduct cognitive testing with 10 patients with SMI prior to undertaking the main study. Cognitive testing entails administration of questionnaires to the target population of a study to investigate whether the respondents understand the questions correctly and give accurate answers. The goal is to modify the questionnaires to versions that capture the research intent and participants understand correctly [46, 47]. Cognitive testing will be done in the language preferred by the participants between English and Setswana.

#### Assessment of exposure to traumatic events

The Childhood Trauma Questionnaire and the Life Events Checklist which have been described under phase 1 methodology will be used to assess exposure to traumatic events.

#### Assessment of post-traumatic stress disorder

*The Post Traumatic Checklist-5* (*PCL-5*) [48]: A self-report measure used for assessment of PTSD. The PCL-5 comprises of 20 items which correspond with 20 DSM-5 symptom criteria for PTSD. Respondents indicate how much they are bothered by each symptom on a 5-point Likert scale ranging from zero (not at all) to four (extremely). The instrument can be used to make a provisional diagnosis of PTSD, screen for PTSD and assess symptom change during treatment which makes it suitable for the current study. The PCL-5 has demonstrated factorial validity and reliability for research use in the South African context [49].

*The Mini International Neuropsychiatric Interview* (*MINI*): Is a short diagnostic structured interview developed to explore 17 psychiatric disorders [50]. The MINI will be used to make a diagnosis of PTSD. The MINI has demonstrated good sensitivity, specificity, validity and reliability in the assessment of psychiatric disorders [51].

#### Assessment of demographic and clinical information

*Researcher-designed Socio-Demographic Questionnaire*: To collect data on demographics such as age, gender, highest level of education, income and clinical history such as family history of any psychiatric illness and substance use.

#### Assessment of symptoms commonly associated with PTSD and functioning

*Clinical Outcomes in Routine Evaluation-Outcome Measure* (*CORE-OM*): The CORE-OM was originally developed in the UK as an outcome measure to compare effectiveness of interventions across National Health Service sites and across different client populations [52]. It is used to assess psychological distress across four domains: subjective well-being (4 items), symptoms of psychological distress (12 items), life functioning (12 items) and risk to self and others (6 items). The CORE-OM is used as a baseline assessment tool at initiation of therapy and as an outcome measure at the end of therapy; it is a useful instrument in quantifying the degree of therapeutic change post treatment [53]. The CORE-OM has been found suitable for use across primary and secondary mental health care [54]; it has been validated in South Africa [55] and in Kenya [56].

*Patient Health Questionnaire-9* (*PHQ-9*): Is a 9-item instrument used to screen for depression. Respondents endorse frequency of depression symptoms in the preceding 2 weeks on a 4-point scale, ranging from 0 (never) to 3 (nearly every day), for a total score ranging from 0 to 27, with higher scores indicating increased likelihood for major depressive disorder [57]. The PHQ-9 demonstrated good internal consistency and criterion validity in a validation study conducted in Botswana [58].

*Zung self-rating anxiety scale* [59]: Is a 20-item instrument that assesses anxiety levels. The respondent endorses frequency of symptoms of anxiety over the past week on a Likert-type scale ranging from one to four. A raw score of 36 has been set as a cut-off point for clinically significant anxiety [60]. The Zung has been used in Botswana previously and demonstrated good internal reliability with a Cronbach’s alpha of 0.80 [61].

#### Assessment of knowledge about PTSD

*Knowledge of PTSD test* (*KPTSD)* [62]: A 15-item multiple-choice test which was designed for use in a study which evaluated a PTSD psych-educational programme for psychiatric inpatients. The KPTSD measures areas of knowledge targeted by BREATHE such as the definition of trauma, symptoms of PTSD and events that may result in PTSD. Although no psychometric data exists for the measure, KPTSD has been shown to be sensitive to the effects of education about PTSD in clients with SMI [62].

#### Changes in skin conductance

Although breathing retraining is a core component of many evidence based interventions for PTSD [63], measurement of physiological arousal of participants undergoing PTSD treatment is not frequently done; most studies rely on self-reported arousal. Psychophysiological measures of skin conductance variability will be used to assess change in arousal. Physiological arousal results in the skin becoming a better conductor of electricity. Variation in electrical conductance will be measured with the eSense EDA skin conductance device electrodes at multiple timelines during the study and variance will be recorded.

The quantitative measures will be administered by trained research assistants at different times as detailed in Table 1.

**Table 1:** Evaluation of diagnosis, clinical symptoms and functioning

Measure	Time of assessment						
	Pre-intervention		Intervention			Post intervention	
	R_0_	T_0_	Week 1	Week 2	Week 3	Month 1	Month 3
MINI PTSD	X	X			X	X	X
CTQ-SF	X						
LEC-5	X						
PCL-5		X	X	X	X	X	X
KPTSD		X	X	X	X	X	X
PHQ-9		X	X	X	X	X	X
ZUNG		X	X	X	X	X	X
CORE-OM		X			X	X	X
Skin conductance		X	X	X	X	X	

*R*_0_ recruitment, *T*_0_ baseline

### Quantitative and qualitative assessment of feasibility, acceptability and satisfaction for patients in the treatment arm

Table 2 demonstrates timelines for administration of the measures.

**Table 2:** Evaluation of feasibility, acceptability and patient satisfaction

Measure	Time of assessment						
	Pre-intervention		Intervention			Post intervention	
	*R*_0_	*T*_0_	Week 1	Week 2	Week 3	Month 1	Month 3
Satisfaction scale					X		
FIM					X		
AIM					X		
Therapist feedback			X	X	X		
Qualitative interviews					X		

*R*_0_ recruitment, *T*_0_ baseline

#### Assessment of patient satisfaction

*Satisfaction Scale*: The researcher designed a 9-item questionnaire with the input from the intervention developer Professor Kim Mueser [45]. The instrument will be used to evaluate participants’ satisfaction with the treatment and get their views on how to make it more satisfactory.

#### Assessment of feasibility and acceptability

*AIM and FIM*: Weiner and colleagues developed brief and reliable quantitative measures of acceptability, appropriateness and feasibility [64]*.* Each construct is assessed with four items which have been found to be valid and reliable; the measures can be used independently or together. Cut-off scores have not been determined yet; however, higher scores indicate greater feasibility and acceptability.

Semi-structured interviews will be conducted with participants who would have undergone the intervention (those who complete and those who do not) to gather information on their experiences about the treatment and perceptions on its acceptability and feasibility. Individual interviews are appropriate for assessing acceptability and feasibility because they produce more details and offer an in-depth analysis of the participant’s thoughts and feelings [65]. Examples of interview questions include: ‘Can you describe what it was like for you to participate in the treatment?’ with Probes such as ‘What were the best things about the treatment?’, ‘What were the worst things about the treatment?’, ‘Was there anything you found challenging during the intervention process?’, ‘what was challenging about it?’, ‘What went well?’. Participants who drop out will be interviewed at the time of dropping out of the study depending on their availability.

*Therapist feedback*: The therapist, KM will complete a form after each session about things that went well, those that did not go well, things they might want to change and any challenges encountered. The data will be useful in assessing feasibility and acceptability of the intervention from the therapist’s perspective.

### Training procedures

#### Study personnel

The study will recruit research assistants with a minimum qualification of diploma in any health-related course such as nursing or counselling psychology. Research assistants will be expected to assist in the collection of quantitative and qualitative data during the pilot trial. The MINI diagnostic tool for PTSD will be administered by the first author who is a mental health professional. A 2-day training will be conducted on recruitment and consenting procedures. Research assistants will also be trained on the different study collection instruments, their use, administration and scoring.

#### Training and supervision of the psychotherapist

KM, who is the researcher carrying out the work and also a psychiatrist, will be administering the BREATHE intervention. She has attended training conducted by one of the developers of the intervention: Professor Kim Mueser and Dr. Lauren C. Ng. Dr. Ng has conducted extensive work on the BREATHE intervention with varied populations from low- to middle-income settings and is thus a suitable trainer for the current study. Training lasted for a period of 1 week and included observations, role play and case studies.

Supervision and mentorship will be provided throughout the project by way of video calls and weekly email communication.

### Data analysis

#### Qualitative data

We will carry out qualitative interviews with a sample of the participants (patients and mental health care workers). The discussions will focus on understanding participant experiences and explore their perception on the feasibility and acceptability of the BREATHE intervention. Interviews will be transcribed and translated where necessary prior to analysis. We will use an interpretative phenomenological approach (IPA) [66] which lends itself to focus on expanding the subjective experiences of participants through a detailed, in-depth exploration participants’ experiences. We will use IPA to tease out thematic categories relevant to participant experience with the BREATHE intervention as well as assessing issues of feasibility and acceptability.

#### Quantitative data

Despite our sample size, all analyses will be performed according to the ‘intention to treat’ (ITT) methodology [67]. All data collected from enrolled participants will be used in the analysis regardless of their level of participation. All participants will be analysed according to their initially assigned study arm at baseline.

Descriptive statistics will be provided for all normally distributed variables in mean scores and standard deviations for each assessment instrument at all timepoints. The interaction between times and treatment conditions (BREATHE vs. TAU) on our primary and secondary outcome variables will be examined using a repeated measure ANOVA. This analysis will also be repeated at follow-up. Change scores will be calculated by subtracting the scores at baseline from the scores following the intervention.

### Outcomes

#### Primary outcomes

1. Feasibility and acceptability outcomes

A priori minimum thresholds for feasibility and acceptability will be set [68]. Consent and retention rates for patients who attend all 3 sessions will be set at 50% as this is in keeping with dropout rates in studies of PTSD interventions in resource-limited settings [69], and level of satisfaction will be 70% or greater. The following will be used to assess feasibility and acceptability:

i. Consent rates will be calculated by dividing the number of participants who meet the criteria for PTSD and meet the inclusion criteria for the pilot trial by the number of those who consent to participate in the trial. Reasons for not consenting will be documented.
ii. Acceptability will be assessed by dropout/retention rates [70]. Retention/adherence, through all the three sessions, measured by the total number of sessions attended by the participants and reasons for non-adherence. Participants who do not show up or complete only one session will be categorised as dropouts. Completion at least two sessions will be defined as treatment exposure [71].
iii. Completion of homework and use of breathing retraining techniques
iv. Patient satisfaction
v. FIM and AIM instruments [64] and individual qualitative interviews

2. Reduction in severity of PTSD symptoms (exploratory outcome)

i. Assessed immediately after the intervention as assessed by the PCL-5
ii. Reductions in physiological arousal as measured with sensors to assess skin conductance
iii. PTSD diagnosis assessed with the MINI plus PTSD module

3. Knowledge of PTSD measured by using the knowledge of PTSD scale [62]

### Secondary outcomes

1. Reduction in PTSD symptoms measured using the PCL-5 1 month and 3 months post treatment to determine whether effects of the intervention are sustained.
2. Reduction in symptoms commonly associated with PTSD and improved functional status
  - Depressive symptoms as measured with the Patient Health Questionnaire-9 [57]
  - Anxiety symptoms assessed with the Zung anxiety scale [59]
  - Psychological distress assessed with the CORE-OM [52]

## Discussion

There is no national epidemiological data on mental health in Botswana; however, a health statistics report revealed that there has been an increase in both psychiatric in-patient and out-patient attendances from 2002 to 2010 [72].

Although no estimates on the prevalence of the co-occurrence of PTSD and SMI have been published in Botswana, studies conducted elsewhere have reported prevalence rates of PTSD in patients with SMI ranging between 29 and 43% [73]. Anecdotal findings by the authors have revealed that a significant proportion of patients admitted at the referral hospital (with severe mental illness) have a history of exposure to traumatic events and/or post-traumatic stress symptoms. Screening for trauma exposure and PTSD remains overlooked in the treatment of patients with SMI thus limiting the quality of care provided to patients with SMI. This highlights the need for an intervention in this population.

Due to limited specialised psychiatric human resource, there is a need for task sharing in order to meet the demands of patients with SMI. As such interventions need to be easy to understand by nurses or lay workers. A suitable intervention in Botswana should be effective in patients with comorbid severe mental illness and post-traumatic stress disorder and should require no specialised mental health training. The Brief Relaxation, Education and Trauma Healing (BREATHE) intervention is an evidence-based intervention for PTSD in patients with SMI [13]. BREATHE can be administered by providers without specialised mental health training thus suitable for addressing limited mental health human resource in Botswana.

Psychological interventions have been shown not to be culturally sensitive [74]. For the intervention to be effective, it has to integrate cultural influences in the expression of symptoms of PTSD whilst taking into consideration the local definitions and experiences of trauma. This will ensure clinical utility, acceptability and efficacy of the intervention. BREATHE will be the first psychological intervention to be adapted for the culturally diverse Botswana population. Having an intervention which has been adapted and assessed for efficacy amongst the local population will ensure a culturally sensitive holistic approach to the management of patients with comorbid PTSD and SMI, an area which is currently neglected. The findings from the feasibility trial will inform the design of the future efficacy trial.

## Trial status

This is version 1 of the protocol. The trial is registered on clinicaltrials.gov. Data collection for phase 1 is ongoing. Any deviations to this protocol will be submitted to the respective ethics boards and updated on clinicaltrials.gov, and the changes will be discussed on dissemination of the results.

## Data Availability

Not applicable

## Abbreviations

AIM: Acceptability of Intervention Measure
BREATHE: Brief Relaxation, Education and Trauma Healing intervention
CORE-OM: Clinical Outcomes in Routine Evaluation-Outcome Measure
CTQ-SF: Childhood Trauma Questionnaire-Short form
DSM V: Diagnostic statistical manual fifth edition
FIM: Feasibility of Intervention Measure
LEC-5: Life Events Checklist for DSM-5
MINI: Mini International Neuropsychiatric Interview
PCL-5: Post Traumatic Checklist-5
PTSD: Post-traumatic stress disorder
SMI: Severe mental illness
WHO: World Health Organisations
